# Personality, Behavior Characteristics, and Life Quality Impact of Children with Dyslexia

**DOI:** 10.3390/ijerph17041415

**Published:** 2020-02-22

**Authors:** Yanhong Huang, Meirong He, Anna Li, Yuhang Lin, Xuanzhi Zhang, Kusheng Wu

**Affiliations:** 1 Mental Health Center, Shantou University Medical College, North Taishan Road, Shantou 515065, China; huang.yh@163.com (Y.H.); 18yxlin@stu.edu.cn (Y.L.); zhangxuanzhi2018@163.com (X.Z.); 2 Department of Preventive Medicine, Shantou University Medical College, Shantou 515041, China; st_hemeirong@163.com (M.H.); st_lianna@163.com (A.L.)

**Keywords:** children, dyslexia, risk factors, personality, behavior problem, life quality

## Abstract

Dyslexia is one of the most common neurobehavioral disorders. Children with dyslexia usually suffer from negative, behavior personality problems, and impacted life quality. We aimed to identify family environment factors for dyslexia, and to evaluate the personality, behavior characteristics and life quality of children with dyslexia. A total of 60 children diagnosed with dyslexia and 180 normal children that were aged 7–12 who speak Chinese were recruited from four primary schools in Shantou City, China. Self-designed questionnaire, children’s edition of the Eysenck Personality Questionnaire (EPQ), Conners’ Parent Rating Scale (CPRS), and Quality of Life scale for children and adolescents (QLSCA) were employed for investigation. Multiple logistic regressions show that antenatal training (*OR* = 0.36), higher household income, higher parents’ educational levels, and parents engaging in white-collar jobs were negatively associated with dyslexia; while, family members also suffering from dyslexia (*OR* = 12.17), lower frequency of communication between parents and children, and worse parent-child relationship were positively associated with dyslexia. Children with dyslexia scored higher in psychoticism and neuroticism (*p* = 0.040, 0.008), but lower in extroversion and dissimulation than normal children (*p* = 0.025, 0.007) in the EPQ test. They tended to be more introversion (68.3% vs. 43.0%), psychoticism (25.0% vs. 13.3%), and neuroticism (46.7% vs. 18.8%) than the controls. In addition, children with dyslexia had higher scores in conduct problem, learning problem, hyperactivity, and Conners’ index of hyperactivity (CIH) in CPRS test; and, lower scores of psychosocial function, physical and mental health, and satisfaction of living quality in QLSCA test (all *p* < 0.05). Several family environment and parenting factors were associated with children’s dyslexia significantly. Children with dyslexia had the personality of psychoticism, neuroticism, introversion, and more behavioral problems. Dyslexia significantly impacted the children’s quality of life. Our findings provide multiple perspectives for early intervention of dyslexia in children, particularly in family factors and the parenting environment.

## 1. Introduction

Dyslexia is a specific learning disorder that stems from the lack of ability to decode words and it is usually reflected in the ability to process sounds. This ability to decode is not related to intelligence, age, sensory abilities, other cognitive abilities, or general developmental disorders [1]. Dyslexia is manifested by difficulties inaccurate word recognition and poor performance in reading and writing. It is one of the most common neuro-developmental disorder in children. Approximately 5–10% of school-age children suffer from dyslexia, which is more common in boys [2]. The prevalence of dyslexia in Hong Kong is about 9.7% to 12.6%, according to a 2007 survey [3]. An epidemiological study on the children with Chinese developmental dyslexia was performed in Guangzhou, China in 2019, the reported prevalence rate was4.9%, boys had a higher rate of dyslexia than girls (*OR* = 4.17) [4].

The etiology and pathogenesis of dyslexia have not yet been clearly determined. At present, three views dominate explanations of dyslexia: A visual-attentional view, a phonological view, and a multifactor view [5]. Scholars have found that children with dyslexia may be related to genetic, brain injury, brain dysplasia, malnutrition, and so on [6,7]. Children with dyslexia lag behind normal children in visual cognition and sequential motor skills [8], and children’s learning motivation is also an important cause of dyslexia [9]. External factors, including school, family environment, parenting education and reading environment, living environment, and others may also influence children reading skills. Children’s living and learning environment has produced significant effect on their learning skills [10]. Incomplete family, disharmonious family relationships, family dysfunction, and too harsh parental rearing patterns may cause children anxiety or resist in learning [11].

The life quality of children with learning disabilities is worse than that of typical development children, and the severity of learning disabilities is positively correlated with a poor quality of life [12]. Health-related life quality of children with dyslexia has been damaged, covering several social psychology function (including the role/social restrictions that are caused by emotional problems, general behavior, and mental health) and somatic sensation (including somatic function, the social limits that are caused by somatic health, general health perception). These damages mainly manifest as poor social intercourse ability, low level of peer acceptance and self-awareness, more negative emotion and behavior problems, and a high juvenile crime rate [11,13,14]. The researchers reported that learning disabilities accounted for a large proportion in juvenile delinquency [15,16]. Children with dyslexia have negative emotions on self-image, peer and family relationships, and social interaction. As for social interaction, children with dyslexia lack social skills due to pressure or low self-esteem, and they have many problems with social adaptive behaviors and personality. The incidence of anxiety and depression in children with dyslexia was also higher than that in normal children, with more negative behaviors, higher suicide rate, and increased antisocial behaviors [17]. 

Dyslexia has become an important issue affecting the cognitive, personality development and mental health development of school-age children. Children with dyslexia have low academic scores and unstable emotions, and often feel inferior due to the incomprehension of their parents, teachers, classmates, and peers. If things continue this way, they may even suffer from anxiety and depression, which have adverse effects on their physical and mental health. To date, several studies provide evidence regarding the effectiveness of a literacy intervention programme on enhancing learning outcomes for children with dyslexia [18,19]. However, dyslexia is associated with multiple factors, and interventions that focus on improving linguistics-literacy may not be enough. Therefore, it is important to understand the behavior and personality characteristics of children with dyslexia and explore the factors that are related to dyslexia. 

The purpose of this study was to investigate the family environment factors of dyslexia, the relationships between children’s personality traits and dyslexia, and the possible behavior and life quality impairments. We also aim to provide clues for future effective prevention and intervention of dyslexia in children based on the family environment factors investigation in order to reduce the severity of symptoms in children with dyslexia.

## 2. Materials and Methods 

### 2.1. Study Participants

In this study, the case group was children with dyslexia that were diagnosed in Mental Health Center, Shantou University Medical College, who were school-age children from four primary schools in Shantou city in 2018. The control group was typical development children of the same age and grade from the same four primary schools. The recruited children have been finally diagnosed as dyslexia according to Diagnostic and Statistical Manual of Mental Disorders, fifth edition (DMS-5) and confirmed by literacy tests by experts from the Mental Health Center of Shantou University Medical College [20]. Our research group compiled the literacy test tool (40–50 min), named Assessment of Chinese Literacy for Primary School Students (ACLPSS), in previous screening survey. The assessment tool includes the following five subscales: Phonological awareness, Morphological awareness, Rapid automatized naming, Orthographic processing, and Reading ability subscales. Criteria for case group inclusion: (1) Intelligence test ≥85; (2) Literacy is more than 1.5 standard deviation below the average of normal children of the corresponding grade; (3) Having difficulty in learning and using academic skills for six months and reaching symptom criteria; (4) Children with dyslexia strictly diagnosed by psychiatrists after literacy tests; (5) Grade 2–5 and aged from seven to 12. Criteria for inclusion of the control group: grades 2–5, aged 7–12, normal children without dyslexia. Children with intelligence problems or visual and auditory problems, neuro-developmental deficiency, color blindness, mental deficiency, and the rejection of participants in both groups were excluded from the study.

For children who met the inclusion criteria, general information was collected by questionnaire after they and their parents signed the informed consent form, and then the specially trained psychiatrist conducted the following tests. The case group and control group were anonymously investigated. The investigators explained the purpose, significance, filling method, and announcements of the investigation to the subjects before formal investigation, in accordance with uniform methods and standards. The survey was conducted on a one-to-one basis, in which an investigator surveyed a child and his/her parents and then instructed them to fill out the questionnaire but avoiding inducement. The investigators carefully examined the questionnaires for the possibly missing or incorrect information after collecting them. The ethics committee of Shantou University Medical College approved the survey and written informed consents were obtained from participants and guardians prior to the investigation.

### 2.2. Instruments

General demographic and family characteristics: The self-designed general information questionnaire was applied to obtain the basic demographic information and family characteristics, covering age, gender, grade, parental educational levels, parental occupation, family income, whether single-parent family, parental rearing pattern, antenatal training (antenatal training means that the fetus can constantly appreciate music, poetry, and story recitation in the womb), study and training before age three, frequency of communication between parents and children, sleep status of children, parent-child relationship, and so on. The parents answered the questionnaire.

EPQ: The Eysenck Personality Questionnaire (EPQ)-children version revised by Yaoxian Gong in China [21] was used to evaluate the personality of the investigated children. EPQ includes 88 items, which can be divided into four dimensions: P (Psychoticism), E (Extroversion/Introversion), N (Neuroticism), and L (Dissimulation). The children version of EPQ is applicable to children that were aged 7–12 in this study. 

The standard score T (T = 50 + 10 × (x − m)/SD) can be converted from norms to analyze the personality characteristics of the subjects, according to the total score (rough score) that was obtained by the subjects on each scale. T scores between 43.3 and 56.7 points in each scale were of the intermediate type; T scores between 38.5 and 43.3 points or between 56.7 and 61.5 points were of the propensity type; and, T scores below 38.5 points or above 61.5 points were typical.

CPRS: Conners’ Parent Rating Scale (CPRS) was used to assess children’s behavioral problems by the parents. There are 48 items in the questionnaire, and the four-level scoring method (0, 1, 2, 3) could be summarized into six factors, including conduct problem, learning problem, somatic complaints, hyperactivity, anxiety, and CIH (Conners’ Index of Hyperactivity). A factor score that was higher than 1.5 indicates an abnormality (or a deviation of 2 standard deviations). CPRS basically summarized the common behavior problems of children, and their reliability and validity have been tested. This scale has a Cronbach’s α of 0.923 in the current study.

QLSCA: The Quality of Life scale for children and adolescents (QLSCA) was used to evaluate the life quality of the dyslexia children. It was developed in 2000 by Tongji Medical College of Huazhong University of Science and Technology in China, and then revised in 2002 [22]. There are 49 items in the scale, which can be summarized into four factors (aspects) with 13 dimensions: the social psychological function (including 21 items covering teacher-student relationship, peer relation, parent-child relationship, learning ability and attitude, and self-concept); physiological and psychological health (including 12 items covering somatic sensation, negative emotions, attitude of homework); living environment (including eight items covering life convenience, extracurricular activity, athletic ability), and quality of life satisfaction (including self-satisfaction and others). Dimension score = the sum/number of items that make up a dimension; and, factor score = the sum/number of dimensions that make up a factor. Low scores indicate poor quality of life, while high scores indicate good quality of life. Each item is scored from 1 (never), 2 (seldom), 3 (often), and 4 (always). The children approved the QLSCA. The self-report method was used to evaluate the score level, and all of the questionnaires were assessed strictly according to the prescribed procedures and the standard scoring method. The questionnaire has a Cronbach’s α of 0.90 in this study.

### 2.3. Statistical Methods

All of the data were checked and input with Epidata3.1 (The EpiData Association, Odense, Denmark) and Excel 2016 (Microsoft Corporation, Redmond, WA, USA) software. The data analyses and graphing were performed in SPSS 24.0 statistical software (IBM Corporation, Armonk, NY, USA) and GraphPad Prism 8.0 (GraphPad Software, Inc., San Diego, CA, USA). The normal distribution of the data was verified by using of Kolmogoro-Smirnov and Shapiro-Wilk statistics. The data were presented as mean ± SD for continuous data and number (percentages) for categorical data. Independent sample *t* test or chi-square tests were used to analyze the differences of general and personality characteristics, behavior problems, and life quality between dyslexia and normal children. Univariate and multivariate Logistic regression models were used to explore the influencing factors for dyslexia. A two-sided of *p* < 0.05 was considered to be statistically significant.

## 3. Results

### 3.1. General Characteristics of the Participated Children

A total of 60 children with dyslexia and 180 normal children were finally included in this study and they finished all of the questionnaires and tests (Table 1). There was no difference of age, sex, or residence between children with dyslexia and normal children. The proportion rates of children from only-child family and single parent family were not different between the two groups.

### 3.2. Family Environment, Parenting Education and Children’s Dyslexia

Children with dyslexia received less antenatal training than the control group (16.7% vs. 35.6%, *p* = 0.006). While no difference of preschool education before age of three years or time to start speaking existed between the two groups. The children with dyslexia had more family members also suffering from dyslexia when compared with normal children (*χ*^2^ = 22.981, *p* < 0.001). The parents had difference in the frequency of communication with their children in the case and control group (*χ*^2^ = 14.631, *p* = 0.002). The communication frequency was lower between the children with dyslexia and their parents when comparing with the normal children and their parents. Parent-child relationship was fewer in “good” scale, but more in “general” or “bad” scale in children with dyslexia as compared to normal children (*χ*^2^ = 6.142, *p* = 0.046). Parents’ occupation and education levels were all statistically different between the two groups (all *p* < 0.05). The parents of the children with dyslexia tended to be engaged in farming and physical labor, and they had relatively lower education levels. The monthly household income was lower in the dyslexia group than in the control group (*χ*^2^ = 7.890, *p* = 0.019, Table 1). 

Univariate and multiple logistic regression models were performed in order to evaluate the family environment factors associated with dyslexia. The results that were based the multiple logistic regression models showed that antenatal training (*OR* = 0.36, 95% CI: 0.17–0.76), higher parents’ education levels, parents engaging in white-collar jobs, and higher monthly household income were negatively associated with children’s dyslexia; while, family member also suffering from dyslexia (*OR* = 12.17, 95% CI: 3.79–39.05), bad parent-children relationship (*OR* = 6.68, 95% CI: 1.18–37.71), and low frequency of communication between parents and children (*OR* = 9.71, 95% CI: 1.88–50.12 for “less than 1 time per week” as compared to “every day”) were positively associated with children’s dyslexia (Table 2).

### 3.3. Personality Characteristics of Children with Dyslexia

The Eysenck Personality Questionnaire (EPQ) was used to evaluate the personality characteristics of the subjects. The results showed that children with dyslexia and normal children had statistical differences in personality characteristics, including dimensions of Psychoticism, Extroversion/Introversion, Neuroticism, and Dissimulation (all *p* < 0.05). Children with dyslexia had higher scores than normal children in the two dimensions of Psychoticism and Neuroticism; while, lower scores than normal children in the dimensions of Extroversion/Introversion and Dissimulation (Table 3), without gender difference (data not shown).

**Figure 1:**
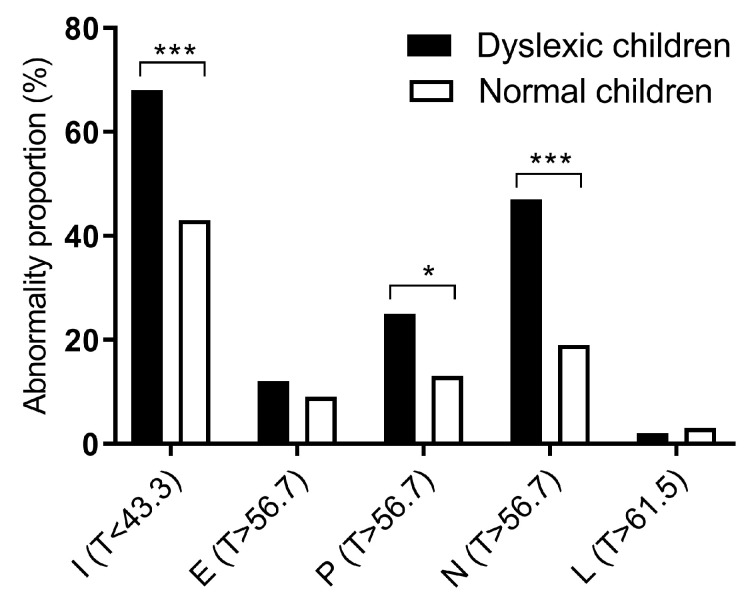
The abnormality proportion of Eysenck Personality Questionnaire (EPQ) scores between the dyslexia and control groups. E is Extroversion, I is Introversion, P is Psychoticism, N is Neuroticism, and L is Dissimulation. T is the standard score calculated by the formula: T = 50 + 10 × (x − m)/SD. *N* = 240. * *p* < 0.05, *** *p* < 0.001.

The abnormality proportions of the personality characteristics of children with dyslexia and normal children were also calculated based on the T scores. The results showed that the abnormality proportions of I (T < 43.3), P (T > 56.7), and N (T > 56.7) in children with dyslexia were all higher than in the normal children (Figure 1). Children with dyslexia were more prone to introversion than normal children (*χ*^2^ = 10.518, *p* = 0.001). They also tended to have more abnormal psychoticism and neuroticism than normal children (*p* = 0.046, *p* < 0.001, respectively). No significant difference of abnormal E (T > 56.7) or L (T > 61.5) was found between the two groups.

### 3.4. Behavioral Characteristics of Children with Dyslexia

**Figure 2:**
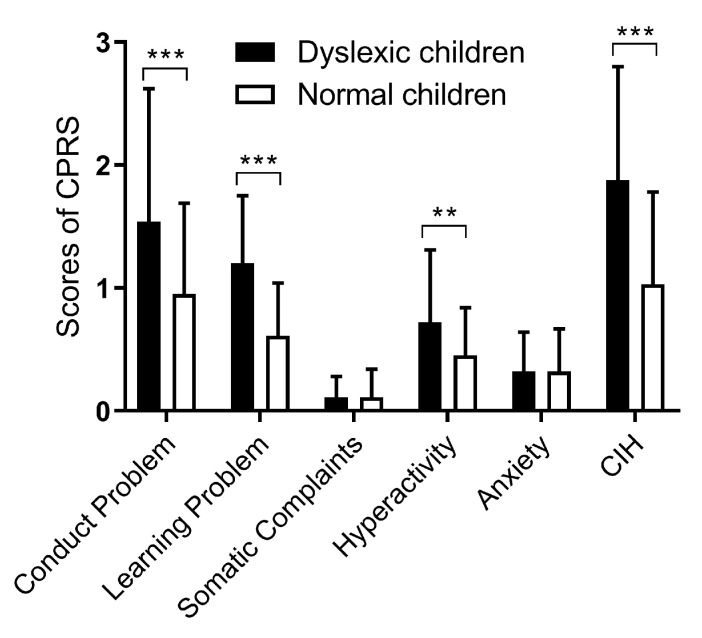
Comparison of Conner’ Parent Rating Scale (CPRS) scores between the dyslexia and control groups. *N* = 240. ** *p* < 0.01, *** *p* < 0.001. CIH, Conners’ Index of Hyperactivity.

CPRS was applied to evaluate the behavior characteristics of the participated children based on the parental answers. The results indicated that children with dyslexia had higher scores in the dimensions of conduct problem, learning problems, hyperactivity, and Conners’ index of hyperactivity (CIH) than the normal children (all *p* < 0.01), which means that children with dyslexia had more behavioral problems. No statistically significant difference of somatic complaints and anxiety was found between the two groups (Figure 2).

### 3.5. Life Quality of Children with Dyslexia

QLSCA was used to assess the life quality of the participated children. We found that children with dyslexia had lower scores in the total score and eight dimensions of QLSCA, including: teacher-student relationship, peer relation, parent-child relationship, learning ability and attitude, somatic sensation, attitude of homework, life convenience, and self-satisfaction when compared with the normal children; but, had a higher score in the dimension of extracurricular activities than normal children (all *p* < 0.05, Table 4). There was no statistical difference in the dimensions of self-concept, negative emotion, athletic ability, and others (all *p* > 0.05).

The thirteen dimensions of QLSCA were further constituted four factors of life quality, including psychosocial function, physical and mental health, living environment, and satisfaction of living quality. The results suggested that the scores of psychosocial function and physical and mental health were both lower in children with dyslexia than in normal children (both *p* < 0.001, Table 4). There was no statistical difference in the scores of living environment and the satisfaction of living quality between the two groups in this study.

## 4. Discussion

In this case-control study, family environmental factors, personality characteristics, behavioral characteristics, and life quality of dyslexia children were investigated. Our results suggested that children’s dyslexia was associated with genetic and family environment factors, as well as antenatal training, parents’ education and occupation, monthly household income, etc. Children with dyslexia tended to have more negative personality of introversion, psychoticism, and neuroticism, and they also had more behavior problems with unstable emotions. Their life quality was impacted to some extent.

### 4.1. Family Environment, Parenting Factors and Dyslexia

Family environment is of special significance to children’s learning initiation and development [23]. We found, in this study, that the parents of children with dyslexia were less educated than those of normal children, and engaged in relatively poor jobs. Higher levels of education are often closely related and consistent with better jobs, and better household income. Additionally, the family of children with dyslexia had relatively lower monthly household income in this study. Parents are the first teachers of children, and their education plays a vital role in the growth of children’s learning. Well-educated parents usually had children better than their peers in many aspects of language development and learning ability [24]. The higher the education level of parents, the easier it is for them to raise high-quality children [4]. In addition, parents with higher education levels and who are white-collar employees pay more attention to exchange and communication with their children, thus the parent-child relationship is relatively close, which is also observed in this study. When dealing with children, they can persuade and educate in a milder way, rather than scolding or criticizing. The higher the parent’s education levels, the better the family education atmosphere is, and the more the reading experience the children will have before age of six years [4]. Some studies have found that parents with low education level pay more attention to their children’s academic performance, while the parents with high education level pay attention to all aspects of children’s physical and mental development, and the parent-child relationship quality affects young children’s physical and mental health [25,26]. In addition, dyslexia is genetic determined. Lower parental literacy stimulation might result from parents being dyslexic. As presented in our results, there is higher proportion of parents and other family members with dyslexia in the case group.

Interestingly, antenatal training was found to negatively correlate with dyslexia in this study, which needs further studies to confirm. Antenatal training such as music appreciation, listening poetry and story recitation may promote children’s learning and language skills. Prenatal education especially music and language education can help to reduce the incidence of behavior problems in preschool children [27]. In the gestation period, the fetal brain function, hearing, feeling, and so on all obtained the initial development due to antenatal training. The edification is beneficial in the growth and development of the fetus. Antenatal training through both music and maternal talk to the unborn fetus might reduce the risk of children’s autistic-like behaviors [28]. Children with early education and training developed language skills well, and they had a clear appreciation for understanding and listening to speeches [29,30]. 

We found that children with dyslexia and their parents less frequently communicated than normal children. Some psychologists have pointed out that parents are the most important playmates of their children. In the process of growth and development, if parents rarely communicate with their children intimately, it is very easy to cause children’s personality defects and make them feel insecure [31]. The generation of negative emotions, such as loneliness and sensitivity, caused the child’s emotional control ability to deteriorate, thus affecting the child’s personality development [32]. In addition, communication between parents and children can promote the development of children’s language skills [33]. Adequate communication can also promote a child’s language organization. The weak organizational ability of the language system is also one of the characteristics of children with dyslexia. Therefore, good communication is essential for children’s language learning and growth. Parents’ criticizing, neglecting, blaming, and other negative educational methods will be not conducive to children’s learning and growth.

### 4.2. Children with Dyslexia and Their Personality and Behavior Characteristics

Children show different personality characteristics from birth, and personality refers to the stable and consistent behavioral tendency, and the emotional and behavioral response patterns in different situations. Personality traits are determined by heredity and influenced by surrounding environment. Positive emotions can improve learning efficiency and promote learning, while a negative mood can hinder learning. In this study, children with dyslexia score significantly higher in Psychoticism of EPQ test, which indicates that they tend to have high traits of psychoticism. When compared with normal children, some children with dyslexia tend to be eccentric, withdrawn, and troublesome. Such children have a profound lack of sense of right and wrong and often do things without regarding safety. They have no concept of socialization, being characterized by a tendency toward psychoticism.

Our results suggest that children with dyslexia were more prone to show emotional instability, which is, tended to neuroticism. Children with dyslexia scored higher in neuroticism of EPQ test than children without dyslexia. They tended to feel more anxious in emotional aspects, tended to be easily nervous when things happened, had short temper and irritability, and tended to have depression. They might often suffer from poor sleep, psychosomatic disorders and emotional distress, and might respond too strongly to stimuli that are difficult to calm down. Because the strong emotional reaction often affects their normal reaction, usually expressed as being unreasonable, and sometimes even in a dangerous way. Emotional instability is more likely to lead to their lack of concentration, often anxious, or even depression, leading to an inability to concentrate on learning or insufficient learning ability.

Children with dyslexia tended to be introversion in this study. They were often shy, quiet, aloof, and introspective in their study and life. They spoke little and did not like to contact with others. The social interaction approach to language development emphasizes the importance of face-to-face communication in early language development and the importance of guiding the adjustment of children’s language and language ability [34]. Children who are highly social and extroverted are more likely to find interacting partners and seek out more opportunities to communicate. On the contrary, shyness limits children’s social contact. Shy children spend less time interacting with unfamiliar peers or adults in new situations, and they receive less stimulation and practice in language skills than their peers. Thus, introversion or extroversion can be considered as different characteristics that affect language learning in social interaction. There are a large number of studies that support the view that social interaction might be beneficial for language development and learning. Studies have proved that introversion children’s vocabulary is less than the normal peers, and extroversion is positively related to the children’s vocabulary [35,36]. Children’s personality traits (especially extroversion and emotional stability) are suggested to relate to children’s vocabulary expression and understanding in another study [37], and emotional stability and extraversion have an overall impact on children’s vocabulary expression.

Children with dyslexia have poor academic performance, and they suffer from low self-esteem, anxiety, and emotional instability due to long-term experience of learning frustration, which further affects their learning motivation and emotional state. On the other hand, children’s personality traits will also cause difficulties in language learning. Negative personality characteristics can make symptoms of dyslexia becoming more severe. Dyslexia and personality traits interact and influence each other in learning. Therefore, for children with dyslexia, attention should be paid to behavioral and psychological intervention, to correct for extreme personality and guide them to the healthy development.

Many studies have shown that children with dyslexia have more behavioral problems [38]. This study found that dyslexia children had higher scores of conduct problems, learning problems, hyperactivity, and CIH than those of normal children in the CPRS test. Conduct problems are often prone to conflict with others, such as disobedience to discipline, temper tantrums, sabotage, or theft. Students with these problems are often scolded by teachers and parents, and rejected by classmates, which might easily cause emotional problems and affect their study. Most delinquent youths in their study suffer from dyslexia [39,40], and dyslexia patients tend to have more negative behaviors, such as discipline violation and poor social interaction. Children with dyslexia are often lack of concentration and easily distracted, with more learning problems, and they are often accompanied by hyperactivity. Approximately12–24% of children with dyslexia suffered from another co-occurring neurodevelopmental disorder, namely attention-deficit/hyperactivity disorder (ADHD) [11], which was consistent with our study, namely 18%. Children with dyslexia are often easy to be stimulated by the outside world in class, homework, or other activities, so that attention is difficult to last, with too much activity and impulsive performance. The dyslexic cases had a higher risk of hyperactivity and inattention (OR = 3.21) in a recent Chinese epidemiology study [4]. Attention disorder and hyperactivity often affect their class learning and reduce the speed and quality of their homework, which results in poor academic performance that is lower than their intelligence should achieve.

### 4.3. Children with Dyslexia and Their Life Quality

Adolescence is the key period of somatopsychic development and social role transformation. Good quality of life plays a certain role in promoting children’s somatopsychic development. Children with dyslexia suffer from academic failure, stress and reading difficulties due to impaired reading ability, which leads to a lack of confidence in themselves, and a certain impact on their quality of life. Children with dyslexia were worse than typical readers in social communication and self-concept and they had more psychological and behavioral problems [41]. Macdonald et al. studied children with dyslexia who had delinquency and crime, and the results showed that, when these children in school could not catch up with the academic progress and began to question his own learning ability, they might have psychological inferiority and lower self-esteem, which often caused their externalizing behaviors and delinquency [42,43]. Poor learning experience, low self-esteem, and emotional problems can lower children’s quality of life.

The results of this study showed that the overall quality of life of children with dyslexia was significantly lower than that of normal children, which was consistent with other studies [17,44]. Children with dyslexia had poorer quality of life than normal children, and the severity of dyslexia was positively correlated with a poor quality of life [45]. In this study, the overall life quality of children with dyslexia was mainly manifested as teacher-student distrust, peer tension, parent-child estrangement, poor learning ability, improper learning attitude, possible somatosensory disorder, and often accompanied by anxiety or depression and other adverse emotions. Children with dyslexia scored lower in the dimension of Teacher-student relationship in this study. They felt that they were often treated unfairly by their teachers and their poor academic performance was often ignored by their teachers, which leads to a lot of distrust in the teacher-student relationship. Children with dyslexia also scored lower in the dimension of Peer relation. Humphrey et al. reported that 50% of children with dyslexia were often mocked and bullied by their peers, or even not accepted by peers due to their poor reading ability and poor academic performance [46]. Peer relationship will also have an impact on their self-esteem, and a poor peer relationship is considered to be the influencing factor of low self-esteem in children with dyslexia [47]. Children with dyslexia had a poor attitude towards homework, often felt sick about writing homework, and were not willing to take the initiative to do homework. Children with dyslexia were less satisfied with their sleep, health, friends around them, and their current life conditions, and their social interaction and mental health were worse than normal children. However, the extracurricular activities of children with dyslexia were higher than that of normal children in this study, which can be explained by the fact that most children with dyslexia have impulsivity and hyperactivity. The high extracurricular activities indicated that they were more hyperactive, which resulted in poor concentration. A poor quality of life often impacts their study and life to varying degrees. Researchers also found that dyslexia children’s behavior problems led to their bad social activities; and, their behavior, mental health, physical function, general health perception, and other health-related quality of life were significantly lower than normal children [12], often being accompanied by the feeling of anxiety and depression, and severe limits in the school or friends’ activities.

## 5. Conclusions

Many family environment factors, such as parent-children relationship, communication frequency between parent and children, parent’s education levels and occupations were associated with dyslexia as well as heredity. Children with dyslexia had the personality characteristics tendency of psychoticism, neuroticism, introversion, emotionally unstable, and more behavioral problems. Dyslexia also impacted the children’s quality of life. Although dyslexia is a genetically related disorder, a good family environment and good parenting and reading environment will reduce the severity of symptoms in children with dyslexia, as well as their behavior problems, and will improve their quality of life. In the intervention of children with dyslexia, we should pay more attention to family environment factors, behavior problems, their extreme personality, and guide them to healthy development.

## Figures and Tables

**Table 1:** General characteristics of the study participants.

Characteristics	Dyslexia Group (*n* = 60)	Control Group (*n* = 180)	*χ*^2^/*t*	*p*
Age (Mean ± SD)	9.93 ± 1.01	10.02 ± 0.78	−0.716	0.475
Gender, *n* (%)				
Male	40(66.7)	117(65.0)	0.055	0.814
Female	20(33.3)	63(35.0)		
Only child family	10(16.7)	41(22.8)	1.004	0.316
Single parent family	3(5.0)	15(8.3)	0.721	0.396
Residence				
Urban	52(86.7)	138(76.7)	2.728	0.092
Rural	8(13.3)	42(23.3)		
Antenatal training Preschool education before 3 years	10(16.7) 21(35.0)	64(35.6) 60(33.3)	7.528 0.056	0.006 0.813
Family member also suffer from dyslexia	13(21.7)	4(2.2)	22.981	<0.001
Parent-child relationship				
Good	44(73.3)	147(81.7)	6.142	0.046
General	12(20.0)	31(17.2)		
Bad	4(6.7)	2(1.1)		
Communication between parents and children				
Every day	41(63.3)	123(68.3)	14.631	0.002
33–34 times a week	4(6.7)	29(16.1)		
1–2 times a week	9(20.0)	26(14.5)		
<1 time a week	6(10.0)	2(1.1)		
Time to start speaking				
0.5–2.5 years old	50(83.3)	154(85.6)	0.193	0.908
2.5–4 years old	9(15.0)	23(12.8)		
More than 4 years old	1(1.7)	3(1.6)		
Father’s occupation				
Farmer	12(20.0)	11(6.1)	12.911	0.005
Worker	22(36.7)	56(31.1)		
Businessman/service providers	18(30.0)	69(38.3)		
Professionals/public official	8(13.3)	44(24.5)		
Mother’s occupation				
Farmer	11(18.3)	9(5.0)	14.383	0.002
Worker	23(38.3)	61(33.9)		
Businessman/service providers	24(40.0)	87(48.3)		
Professionals/public official	2(3.4)	23(12.8)		
Father’s education level				
Elementary school or below	17(28.3)	23(12.8)	9.526	0.023
Junior high school	12(20.0)	61(33.9)		
Senior high school	17(28.3)	56(31.1)		
College and above	14(23.4)	40(22.2)		
Mother’s education level				
Elementary school or below	11(18.3)	25(13.9)	8.353	0.039
Junior high school	26(43.3)	48(26.7)		
Senior high school	12(20.0)	55(30.6)		
College and above	11(18.3)	52(28.9)		
Household income (monthly)				
<3000	5(8.3)	14(7.8)	8.677	0.034
3000–5000	35(58.3)	70(38.9)		
5000–10,000	16(26.7)	64(35.6)		
≥10,000	4(6.7)	32(17.8)		

**Table 2:** Multiple logistic regression analysis of the family influencing factors associated with dyslexia in children (*n* = 240).

Investigated Factors	*β*	SE	Wald	*p*	*OR* (95% CI)
Antenatal training	−1.015	0.380	7.138	0.008	0.36(0.17–0.76)
Family member also suffer from dyslexia	2.499	0.595	17.647	<0.001	12.17(3.79–39.05)
Parent-child relationship					
Good					1.00 (Ref)
General	0.257	0.381	0.456	0.500	1.29(0.61–2.73)
Bad	1.899	0.883	4.628	0.031	6.68(1.18–37.71)
Communication frequency between parents and children					
Every day					1.00 (Ref)
3–4 times a week	−0.806	0.565	2.039	0.153	0.45(1.15–1.35)
1–2 times a week	0.401	0.395	1.031	0.310	1.49(0.69–3.24)
<1 time a week	2.273	0.837	7.370	0.007	9.71(1.88–50.12)
Father’s occupation					
Farmer					1.00 (Ref)
Worker	−0.934	0.252	13.788	<0.001	0.39 (0.24–0.64)
Businessman/service providers	−1.344	0.265	25.777	<0.001	0.26 (0.16–0.44)
Professionals/public official	−1.705	0.384	19.673	<0.001	0.18 (0.09–0.39)
Mother’s occupation					
Farmer					1.00 (Ref)
Worker	−0.975	0.245	15.890	<0.001	0.38(0.23–0.61)
Businessman/service providers	−1.288	0.231	31.199	<0.001	0.28(0.18–0.43)
Professionals/public official	−2.442	0.737	10.976	0.001	0.09(0.02–0.37)
Father’s education level					
Elementary school or below					1.00 (Ref)
Junior high school	−1.626	0.316	26.510	<0.001	0.20(0.11–0.37)
Senior high school	−1.192	0.277	18.534	<0.001	0.31(0.18–0.52)
College and above	−1.050	0311	11.429	0.001	0.35(0.19–0.64)
Mother’s education level					
Elementary school or below					1.00 (Ref)
Junior high school	−0.613	0.244	6.339	0.012	0.54(0.34–0.87)
Senior high school	−1.522	0.319	22.832	<0.001	0.22(0.12–0.41)
College and above	−1.533	0.332	21.908	<0.001	0.21(0.11–0.41)
Household income					
<3000					1.00 (Ref)
3000–5000	−0.693	0.207	11.211	0.001	0.50(0.33–0.75)
5000–10,000	−1.386	0.280	24.599	<0.001	0.25(0.15–0.43)
≥10,000	−2.079	0.530	15.374	<0.001	0.13(0.04–0.35)

**Table 3:** Scores of Eysenck Personality Questionnaires between the two groups (Mean ± SD).

Personality	Dyslexia Group (*n* = 60)	Control Group (*n* = 180)	*t*	*p*
Psychoticism	50.25 ± 12.90	46.33 ± 10.00	2.28	0.040
Extroversion/Introversion	40.42 ± 12.53	44.50 ± 11.05	−2.26	0.025
Neuroticism	54.08 ± 11.33	49.65 ± 10.24	2.68	0.008
Dissimulation	48.17 ± 9.78	52.15 ± 7.68	−2.28	0.007

**Table 4:** Comparison of Quality of Life scale for children and adolescents (QLSCA) Scores between the dyslexia and control groups (Mean ± SD).

Dimensions	Dyslexia Group (*n* = 60)	Control Group (*n* = 180)	*t*	*p*
Psychosocial function (21 items)	2.56 ± 0.47	2.84 ± 0.48	−3.96	<0.001
Teacher-student relationship	2.58 ± 0.69	2.92 ± 0.63	−3.449	0.001
Peer relation	2.83 ± 0.61	3.21 ± 0.61	−4.154	<0.001
Parent-child relationship	2.84 ± 0.73	3.05 ± 0.68	−2.035	0.043
Learning ability and attitude	2.27 ± 0.76	2.64 ± 0.70	−3.347	0.001
Self-concept	2.27 ± 0.64	2.37 ± 0.65	−1.029	0.305
Physical & mental health (12 items)	2.74 ± 0.45	2.99 ± 0.45	−3.751	<0.001
Somatic sensation	2.70 ± 0.47	2.91 ± 0.45	−3.115	0.020
Negative emotion	2.77 ± 0.61	2.79 ± 0.63	−0.271	0.784
Attitude of homework	2.75 ± 0.80	3.28 ± 0.62	−5.135	<0.001
Living environment (8 items)	2.70 ± 0.50	2.72 ± 0.54	0.098	0.922
Life convenience	3.01 ± 0.75	3.23 ± 0.73	−1.984	0.049
Extracurricular activities	2.55 ± 0.76	2.28 ± 0.71	2.439	0.016
Athletic ability	2.55 ± 0.76	2.65 ± 0.75	−0.856	0.393
Satisfaction of living quality (8 items)	2.84 ± 0.59	2.96 ± 0.51	−1.429	0.155
Self-satisfaction	2.94 ± 0.69	3.17 ± 0.56	−2.480	0.014
Others	2.75 ± 0.80	2.75 ± 0.72	−0.066	0.947
Total score (49 items)	131.52 ± 18.05	141.64 ± 18.13	−3.670	<0.001

QLSCA, Quality of Life scale for children and adolescents. The 49 items of QLSCA were further divided into 4 factors and corresponding 13 dimensions.

## References

[1] APA (2013). Diagnostic and Statistical Manual of Mental Disorders (DSM-5^®^).

[2] Goswami U., Wang H.L., Cruz A., Fosker T., Mead N., Huss M. (2011). Language-universal sensory deficits in developmental dyslexia: English, Spanish, and Chinese. J. Cogn. Neurosci..

[3] Habib M., Giraud K. (2012). Dyslexia. Handb. Clin. Neurol..

[4] Cai L., Chen Y., Hu X., Guo Y., Zhao X., Sun T., Wu Y., Li X. (2019). An Epidemiological Study on the Children with Chinese Developmental Dyslexia. J. Dev. Behav. Pediatrics JDBP.

[5] Pennington B.F. (2006). From single to multiple deficit models of developmental disorders. Cognition.

[6] Rüsseler J., Ye Z., Gerth I., Szycik G.R., Münte T.F. (2018). Audio-visual speech perception in adult readers with dyslexia: An fMRI study. Brain Imaging Behav..

[7] Wallace M.T., Stevenson R.A. (2014). The construct of the multisensory temporal binding window and its dysregulation in developmental disabilities. Neuropsychologia.

[8] Marchandkrynski M., Bélanger A.M., Morinmoncet O., Beauchamp M.H., Leonard G. (2018). Cognitive predictors of sequential motor impairments in children with dyslexia and/or attention deficit/hyperactivity disorder. Dev. Neuropsychol..

[9] Mammarella I.C., Chiara M., Francesca P., Filippo G., Claudia G., Cesare C. (2009). Representation of survey and route spatial descriptions in children with nonverbal (visuospatial) learning disabilities. Brain Cogn..

[10] Rodriguez E.T., Tamis-LeMonda C.S. (2011). Trajectories of the home learning environment across the first 5 years: Associations with children’s vocabulary and literacy skills at prekindergarten. Child Dev..

[11] Karande S., Bhosrekar K.M. (2009). Health-related quality of life of children with newly diagnosed specific learning disability. J. Trop. Pediatrics.

[12] Karande S., Venkataraman R. (2012). Self-perceived health-related quality of life of Indian children with specific learning disability. J. Postgrad. Med..

[13] Smolik F., Málková G. (2011). Validity of language sample measures taken from structured elicitation procedures in Czech. Ceskoslovenská Psychol..

[14] Furnes B., Samuelsson S. (2011). Phonological awareness and rapid automatized naming predicting early development in reading and spelling: Results from a cross-linguistic longitudinal study. Learn. Individ. Differ..

[15] Einat T., Einat A. (2008). Learning disabilities and delinquency: A study of Israeli prison inmates. Int. J. Offender Ther. Comp. Criminol..

[16] Kumagami T., Kumagai K. (2014). Measuring adjustment in Japanese juvenile delinquents with learning disabilities using Japanese version of Kaufman Assessment Battery for Children II. Psychiatry Clin. Neurosci..

[17] Karande S., Venkataraman R. (2013). Impact of co-morbid attention-deficit/hyperactivity disorder on self-perceived health-related quality-of-life of children with specific learning disability. Indian J. Psychiatry.

[18] Tam I.O.L., Leung C. (2019). Evaluation of the effectiveness of a literacy intervention programme on enhancing learning outcomes for secondary students with dyslexia in Hong Kong. Dyslexia.

[19] Tilanus E.A.T., Segers E., Verhoeven L. (2019). Predicting responsiveness to a sustained reading and spelling intervention in children with dyslexia. Dyslexia.

[20] Huang Y., Xu C., He M., Huang W., Wu K. (2020). Saliva cortisol, melatonin levels and circadian rhythm alterations in Chinese primary school children with dyslexia. Medicine.

[21] Gong Y. (1986). Eysenck Personality Questionnaire.

[22] Chen L., Wu H., Mai J. (2007). Comparison of Quality of Life among Primary and Secondary School Students between Urban and Rural in Beijing and Guangzhou. Chin. J. Soc. Med..

[23] Zhang Y., Li H., Zou S. (2011). Association between Cognitive Distortion, Type D Personality, Family Environment, and Depression in Chinese Adolescents. Depress. Res. Treat..

[24] Coplan R.J., Armer M. (2005). Talking Yourself out of Being Shy: Shyness, Expressive Vocabulary, and Socioemotional Adjustment in Preschool. Merrill Palmer Q..

[25] Van Roy B., Groholt B., Heyerdahl S., Clench-Aas J. (2010). Understanding discrepancies in parent-child reporting of emotional and behavioural problems: Effects of relational and socio-demographic factors. BMC Psychiatry.

[26] Hagan M.J., Roubinov D.S., Adler N.E., Boyce W.T., Bush N.R. (2016). Socioeconomic Adversity, Negativity in the Parent Child-Relationship, and Physiological Reactivity: An Examination of Pathways and Interactive Processes Affecting Young Children’s Physical Health. Psychosom. Med..

[27] Luo B.Y., Xian D.X. (2019). The influence of maternal prenatal education on behavior problems in preschool children. Chin. J. New Clin. Med..

[28] Ruan Z.L., Liu L., Strodl E., Fan L.J., Yin X.N., Wen G.M., Sun D.L., Xian D.X., Jiang H., Jing J. (2017). Antenatal Training with Music and Maternal Talk Concurrently May Reduce Autistic-Like Behaviors at around 3 Years of Age. Front. Psychiatry.

[29] Justice L., Logan J., Kaderavek J., Schmitt M.B., Tompkins V., Bartlett C. (2015). Empirically Based Profiles of the Early Literacy Skills of Children with Language Impairment in Early Childhood Special Education. J. Learn. Disabil..

[30] Bleses D., Jensen P., Hojen A., Dale P.S. (2018). An educator-administered measure of language development in young children. Infant Behav. Dev..

[31] Ying L., Zhou H., Yu S., Chen C., Jia X. (2018). Parent-child communication and self-esteem mediate the relationship between interparental conflict and children’s depressive symptoms. Child Care Health Dev..

[32] Xu W., Yan N., Chen G., Zhang X., Feng T. (2018). Parent-child separation: The relationship between separation and psychological adjustment among Chinese rural children. Qual. Life Res..

[33] Noel M., Peterson C., Jesso B. (2008). The relationship of parenting stress and child temperament to language development among economically disadvantaged preschoolers. J. Child Lang..

[34] Gass S.M., Varonis E.M. (1994). Input, Interaction, and Second Language Production. Stud. Second Lang. Acquis..

[35] Crozier W.R., Badawood A. (2010). Shyness, Vocabulary and Children’s Reticence in Saudi Arabian Preschools. Infant Child Dev..

[36] Salley B.J., Dixon W.E. (2007). Temperamental and Joint Attentional Predictors of Language Development. Merrill Palmer Q..

[37] Coplan R.J., Weeks M. (2010). Shy and soft-spoken: Shyness, pragmatic language, and socio-emotional adjustment in early childhood. Infant Child Dev..

[38] Blum R.W., Kelly A., Ireland M. (2001). Health-risk behaviors and protective factors among adolescents with mobility impairments and learning and emotional disabilities. J. Adolesc. Health Off. Publ. Soc. Adolesc. Med..

[39] Dahle A.E., Knivsberg A.M., Andreassen A.B. (2011). Coexisting problem behaviour in severe dyslexia. J. Res. Spec. Educ. Needs.

[40] Poon K., Ho C.S. (2014). Contrasting deficits on executive functions in Chinese delinquent adolescents with attention deficit and hyperactivity disorder symptoms and/or reading disability. Res. Dev. Disabil..

[41] Humphrey N., Mullins P.M. (2010). Self-concept and self-esteem in developmental dyslexia. J. Res. Spec. Educ. Needs.

[42] Macdonald S.J. (2012). Biographical pathways into criminality: Understanding the relationship between dyslexia and educational disengagement. Disabil. Soc..

[43] Terras M.M., Thompson L.C., Minnis H. (2010). Dyslexia and psycho-social functioning: An exploratory study of the role of self-esteem and understanding. Dyslexia.

[44] Felder-Puig R., Baumgartner M.R., Gadner H., Formann A. (2008). Health-related quality of life in Austrian elementary school children. Med Care.

[45] Karande S., Kulkarni M. (2005). Specific learning disability: The invisible handicap. Indian Pediatrics.

[46] Humphrey N. (2002). Teacher and pupil ratings of self-esteem in developmental dyslexia. Br. J. Spec. Educ..

[47] Humphrey N. (2010). Facilitating a positive sense of self in pupils with dyslexia: The role of teachers and peers. Support Learn..

